# Aplastic anemia: a new complication in the recent mysterious hepatitis outbreak among children worldwide: two case reports

**DOI:** 10.1186/s13256-022-03542-0

**Published:** 2022-11-03

**Authors:** Ali Ghanei-Shahmirzadi, Hamid Reihani, Ali Abbasi-Kashkooli, Fereshteh Karbasian, Seyyed Bozorgmehr Hedayati, Mohammadreza Bordbar, Maryam Ataollahi, Seyed Mohsen Dehghani, Bita Geramizadeh

**Affiliations:** 1 Student Research Committee, School of Medicine, Shiraz University of Medical Sciences, Shiraz, Iran; 2 Department of Pediatric Gastroenterology, Shiraz University of Medical Sciences, Shiraz, Iran; 3 Hematology research Center, Shiraz University of Medical Sciences, Shiraz, Iran; 4 Gastroenterohepatology Research Center, Shiraz University of Medical Sciences, Shiraz, Iran; 5 Shiraz Transplant Research Center (STRC), Shiraz University of Medical Sciences, Shiraz, Iran; 6 Department of Pathology, Shiraz University of Medical Sciences, Shiraz, Iran

**Keywords:** Hepatitis, Aplastic anemia, Outbreak, Hepatitis-associated aplastic anemia

## Abstract

**Background:**

Recently, an unknown hepatitis outbreak among children has concerned many individuals worldwide. These cases are frequently reported, mainly from Europe and other countries. In this study, we present two similar patients, who, to the best of our knowledge, are the first cases reported in the Middle East (Shiraz, Fars Province, Iran). Unlike in similar cases reported up until 30 April 2022, our patients’ hepatitis eventually resulted in aplastic anemia.

**Case presentation:**

In this study, we present cases of two Iranian boys aged 13 and 8 years with hepatitis of unknown origin who developed aplastic anemia in the course of hospitalization.

**Conclusions:**

Hepatitis-associated aplastic anemia is a well-known immune-mediated form of aplastic anemia that we detected in our patients and treated with immunosuppressive therapy. One patient established a satisfactory response to the treatment, but unfortunately, the other was declared brain dead.

## Introduction

Since November 2021, there have been reports of an unexplained hepatitis outbreak in several countries. Hepatitis can occur from various causes, including viral infections, autoimmune disorders, toxins, and medications [1]. In April 2022, 145 children with unknown severe hepatitis were reported in the UK, and nine other cases were reported in Alabama [2, 3]. Furthermore, there have been similar reports from Denmark, Spain, Ireland, and Netherlands [3]. At the same time, two children were referred to our center in Shiraz, Iran with hepatitis manifestations, where further laboratory evaluations ruled out all regular causalities. Besides their hepatitis, we noticed pancytopenia in their complete blood count (CBC) test, which raised suspicion for probable bone marrow disorder. Therefore, a bone marrow biopsy was done, and the results were in favor of aplastic anemia.

Aplastic anemia is a rare but life-threatening condition in which bone marrow failure and hypocellularity result in progressive pancytopenia [4, 5]. Aplastic anemia has been categorized into acquired and inherited types [6]. Acquired aplastic anemia is due to an abnormal immune response triggered by different environmental exposures, including drugs, toxins, and viral infections [6]. It appears that cytotoxic lymphocytes and type I cytokines have a role in autoimmune aplastic anemia, and evidence of low quantity and/or function of T-regulatory cells has been found [7, 8]. Since our patients did not have any history of inherited aplastic anemia or any other risk factor for acquired aplastic anemia, we considered hepatitis to be the underlying cause of their aplastic anemia. Therefore, our diagnosis became hepatitis-associated aplastic anemia (HAAA). HAAA is a well-known immune-mediated form of acquired aplastic anemia in which an acute hepatitis episode results in acute or chronic bone marrow failure accompanied by pancytopenia [9, 10]. HAAA was first mentioned in two cases in 1955 [11, 12], but the number of reports reached more than 200 cases by 1975 [13]. Later on, it was documented in up to 2–5% of aplastic anemia cases in the West [14, 15] and 4–10% of the cases in the Far East [16]. Consequently, owing to the extent of this new hepatitis outbreak among children and considering the rareness of having two patients in such a short period with this infrequent diagnosis in our center, we informed the healthcare providers of other aspects of this new outbreak in the hopes of achieving a faster diagnosis and, therefore, better outcome and prognosis for the patients. We present two cases of HAAA who were referred to our center.

## Case presentation

### Case 1

A 13-year-old Iranian boy came to our pediatric emergency department, a referral center affiliated with Shiraz University of Medical Sciences, with the chief complaint of yellowish skin and two red spots on his right leg. Furthermore, his mother mentioned that he had nosebleeds for a week prior to the admission. He developed icterus 2 months before the admission, and after preliminary laboratory evaluations, which revealed elevated liver enzymes, he was diagnosed with hepatitis. One day before admission, his mother suddenly saw some petechiae-like lesions on his right leg, so she brought the boy to our center. We decided to check CBC and performed liver function tests (LFTs). His preadmission medications included folic acid 5 mg once a day and ursodeoxycholic acid 300 mg twice a day. Physicians had prescribed these drugs because of his previous hepatitis diagnosis. On our primary physical examination, his sclera appeared icteric. He had an ulcer on his lower lip. His lungs were clear, and his heart sounds were normal. On abdominal examination, his liver seemed to be slightly enlarged. He also had evidence of ecchymosis on his right leg.

Laboratory investigations revealed pancytopenia on the first CBC [white blood cell count (WBC) 900/µl, hemoglobin (HB) 7.8 g/dl, platelet count (PLT) 4000/µl]. His liver enzymes were elevated [aspartate transferase (AST) 68 U/L, alanine transaminase (ALT) 174 U/L, total bilirubin 1 mg/dL, direct bilirubin 0.29 mg/dL, gamma-glutamyl transpeptidase 26 U/L] as they had been over the past 2 months. Intending to find the cause of his hepatitis, we checked for common viral hepatitis causes, including hepatitis A virus (HAV), hepatitis B virus (HBV), hepatitis C virus (HCV), cytomegalovirus (CMV), and Epstein–Barr virus (EBV), which were all negative. We checked anti-LKM antibody (Ab), anti-dsDNA Ab, cANCA, and pANCA to rule out the possibility of autoimmune disorders, though we did not find any of them to be positive. We also checked ceruloplasmin and serum copper levels to rule out Wilson’s disease, and neither was in favor of Wilson. We checked coronavirus disease 2019 (COVID-19) immunoglobulin (Ig)G and IgM, which were both negative. We also asked about his past drug history (including herbal drugs) and any potential toxin exposure, but we did not find that could have caused hepatitis. Moreover, we performed chromosome breakage test, which was negative, ruling out Fanconi anemia.

**Fig. 1:**
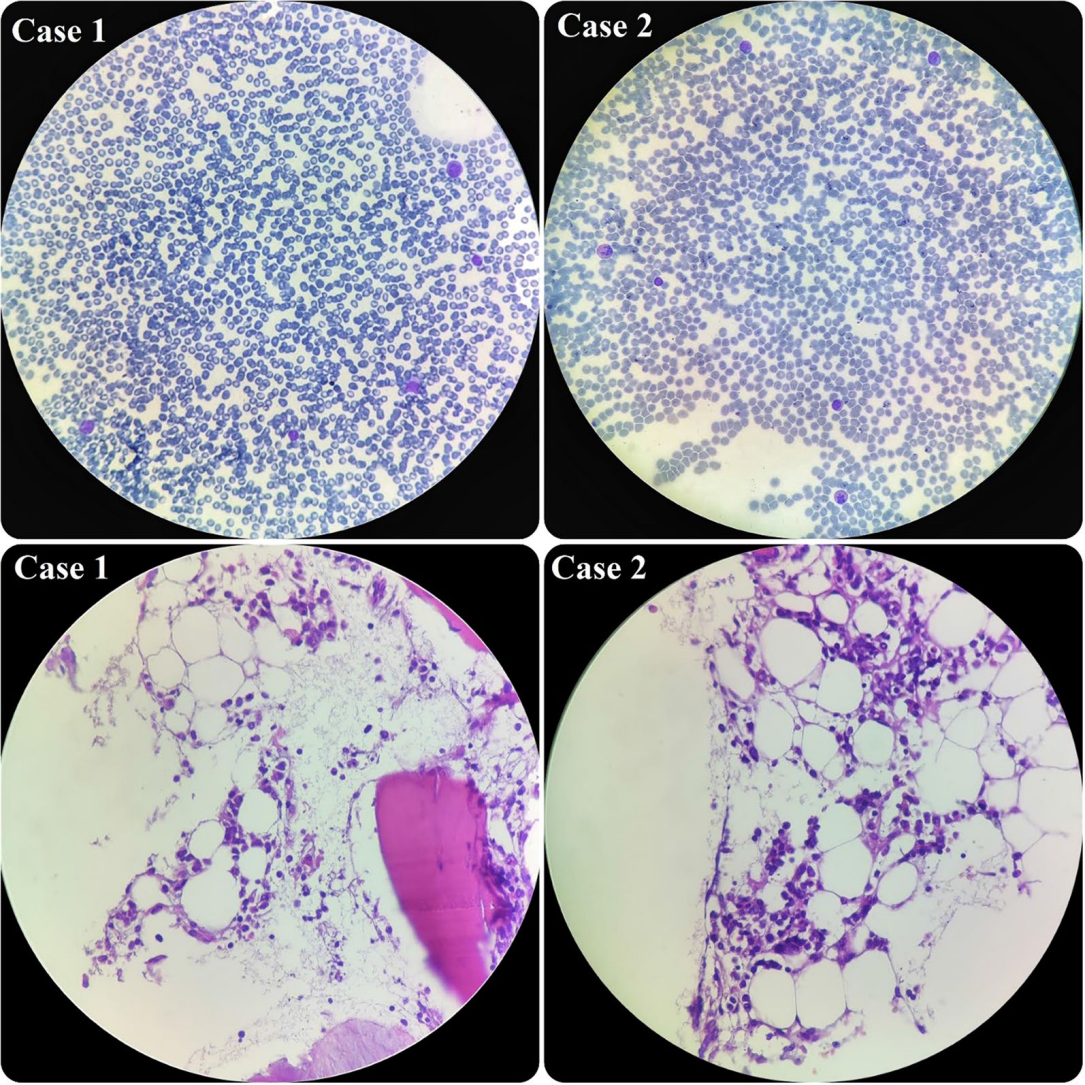
Hypocellular smear from bone marrow aspiration revealing a few scattered myeloid cells and lymphocytes. On bone marrow biopsy, which revealed hypocellularity for his age, intertrabecular marrow spaces were shown to have been replaced by fatty cells beside the presence of some nucleated cells, including lymphocytes, plasma cells, and erythroid cells

In terms of his pancytopenia, we performed bone marrow aspiration and biopsy. The results showed severe hypocellularity (approximately 15%), which was low for his age and suggestive of aplastic anemia. Pictures of bone marrow biopsy and aspiration are shown in Fig. 1. Immunophenotyping by flow cytometry was done on his bone marrow sample, and there was no evidence of leukemia or lymphoma. Our treatment plan was immunosuppressive therapy (IST). The patient was started on equine antithymocyte globulin (ATG) (40 mg/kg/day for 4 days), prednisolone (0.5 mg/kg/day), and cyclosporine (10 mg/kg/day). After receiving treatment, his CBC stabilized (enough to discharge the patient from hospital with further follow-up scheduled) 10 days after IST initiation, and eventually, he was discharged in good condition.

### Case 2

An 8-year-old Iranian boy was brought to our emergency department with the chief complaint of yellowish skin and abdominal distension. He was relatively well until 3 weeks prior to admission. His initial symptoms were anorexia, weakness, and fever, which led him to a medical center. Primary laboratory evaluations were done there, and he was diagnosed with fulminant hepatitis on the basis of elevated international normalized ratio (INR). He also had extremely high liver enzymes. At that medical center, he had received supportive care. Then, since our hospital is the referral liver transplant center in Iran, his parents brought him to our emergency department. On initial presentation, he was severely icteric. His abdomen was distended, and ascites and hepatomegaly were observed.

Our initial laboratory investigations showed severe elevation of his liver enzymes (AST 1615 U/L, ALT 1880 U/L, total bilirubin 47 mg/dL, direct bilirubin 22.8 mg/dL). At that time, he also had coagulation disorder [prothrombin time (PT) 21.1 seconds and INR 2.5]. Moreover, we detected anemia and mild leukopenia in his CBC, but his platelet count was normal (WBC 2900/µl, HB 8.3 g/dl, PLT 190,000/µl). We started searching for the cause of his hepatitis. After checking viral and immunological markers, the only noticeable item we found was a positive COVID-19 IgG Ab. Like the previous patient, he did not report any history of exposure to drugs (including herbal drugs) or hepatotoxic toxins. Moreover, Fanconi anemia was ruled out for the patient by negative chromosome breakage test.

During the admission course, his situation improved and his liver enzymes began to decrease, but he suddenly developed petechiae on his left hand. So, we immediately checked the CBC, which revealed severe pancytopenia (WBC 500/µl, HB 7.1g/dl, PLT 20,000/µl). His platelet count dropped drastically. Therefore, we planned for bone marrow aspiration and biopsy, and the result showed severe hypocellularity, approximately 20%, indicating aplastic anemia. Pictures of bone marrow aspiration and biopsy are shown in Fig. 1.

Since his WBC count was very low, we could not consider a bone marrow transplant, and we decided to try immunosuppressive therapy for him, as the other patient had responded well to it. Unfortunately, after receiving the third dose of ATG, he had an episode of generalized tonic–clonic seizure, and he did not show any response to the treatment until that time. Hence, we stopped the chemotherapy and transferred him to the pediatric intensive care unit (ICU) ward. Unfortunately, he was declared brain dead, due to the low platelet number and coagulopathy. Our patients’ characteristics are presented in Table 1.

**Table 1:** Characteristics of our cases

Characteristic		Case 1		Case 2	
Age (years)		13		8	
Gender		Male		Male	
Chief complaints		Yellowish skin, two red spots on his right leg		Yellowish skin, abdominal distension	
Lab data	AST range		13–68 U/L		130–1615 U/L
	ALT range	27–174 U/L		300–1880 U/L	
	INR	1		1.82–2.82	
	WBC range	900–4000/µl		500–2900/µl	
	HB	7.8–9.3 g/dl		7.1–8.3 g/dl	
	PLT	4000–52,000/µl		190,000–20,000/µl	
Physical examination		Icteric sclera, an ulcer on lower lip, hepatomegaly, ecchymosis on right leg		Icteric sclera, hepatomegaly, ascites	
Bone marrow aspiration		Severe hypocellularity (approximately 15%), suggestive of aplastic anemia		Severe hypocellularity (approximately 20%), suggestive of aplastic anemia	
Outcome		Discharged with good condition		Brain dead	

*AST* aspartate transaminase, *ALT* alanine transaminase, *INR* international normalized ratio, *WBC* white blood cell count, *HB* hemoglobin

## Discussion

Since January 2022, the world has faced an unknown hepatitis outbreak primarily reported in Europe and the USA, mainly in children under 10 years of age [2, 17]. As we have been struggling with COVID 19 during the past 2 years, it is essential to clarify different aspects of this new challenge as soon as possible. Undoubtedly, the most crucial element is to determine the cause, as well as finding proper treatment and identifying the short- and long-term complications of this new hepatitis outbreak. Right now, the leading hypothesis about the source of this outbreak is an adenovirus [2]. However, it is not yet determined whether we are dealing with a new variant or whether the social distancing in these 2 years resulted in fewer exposures to the virus, making children’s naive immune system more susceptible to the previously existing types. It is noteworthy that adenovirus has been known previously to cause acute hepatitis in immunosuppressive children [18].

As of 29 April 2022, according to the World Health Organization (WHO) and UK Health Security Agency (UKHSA), there are at least 200 cases of acute hepatitis of unknown origin that have been reported from 11 countries [2, 3]. Interestingly, 40 out of 53 patients in the UK and 9 out of 9 cases in Alabama that were tested for adenovirus had a positive result [2, 17]. Moreover, the WHO stated that adenovirus had been detected in at least 74 cases [3]. Unfortunately, owing to the lack of laboratory equipment, we could not confirm adenovirus infection in our patients. However, our second case had a positive COVID-19 IgG test. It should be mentioned that, in the majority of confirmed cases, patients were not infected by the COVID-19 virus or vaccinated with COVID-19 vaccines at the time of developing hepatitis. Therefore, we can argue that there is no relation between this outbreak and COVID-19 infection. However, we should consider the high rate of COVID-19 over recent months, especially in children and particularly in England, which has more cases than other countries. This may result in the presentation of a new hepatitis type or mislead us because of its constant presence during this time. Therefore, it is better to perform further experiments to find more substantial evidence.

Severe aplastic anemia usually develops 2–3 months after acute hepatitis attack in patients with HAAA [9]. In our patients, the delay between hepatitis attack and aplastic anemia was close to 2 months as well. In both cases, we started immunosuppressive therapy as soon as the diagnosis of aplastic anemia was confirmed, which consisted of a combination of cyclosporine, ATG, and steroids. Previous studies have shown a 30–70% response to immunosuppressive therapy treatment in children with acquired aplastic anemia [19–21]. We tried the same regimen on our patients, and one of them benefited from this treatment. However, the other patient did not respond well owing to his poor condition, and eventually, he was declared brain dead. Although we used this particular IST regimen in our patients, we recommend to test other accepted regimens as well and compare and analyze the results to identify the best treatment.

## Conclusions

Hepatitis-associated aplastic anemia (HAAA) is a well-known immune-mediated form of aplastic anemia that we detected in our patients. To the best of our knowledge, our study is the first to report the co-occurrence of aplastic anemia with the recent unknown outbreak of hepatitis among children. Therefore, we recommend being alert to hepatitis cases that develop signs and symptoms of pancytopenia, and performing further follow-ups for early diagnosis of potential aplastic anemia.

## Data Availability

Data of the patient can be requested from the authors. Do not hesitate to get in touch with the corresponding author if you are interested in these data.

## Abbreviation

HAAA: Hepatitis-associated aplastic anemia
